# Case Report: Staged topical herbal therapy for giant plasma cell mastitis in pregnancy with 3-year follow-up

**DOI:** 10.3389/fmed.2026.1848671

**Published:** 2026-06-12

**Authors:** Chao Gao, Ruiping Niu, Ke Zhang, Xiaojun Zhang

**Affiliations:** Xiyuan Hospital of China Academy of Chinese Medical Sciences, Beijing, China

**Keywords:** case report, maternal-fetal safety, plasma cell mastitis, pregnancy, topical traditional Chinese medicine

## Abstract

**Background:**

Plasma cell mastitis (PCM) is a benign immune-mediated inflammatory breast disease predominantly affecting non-lactating women. In pregnant patients, conventional treatments carry significant maternal-fetal safety risks, creating an unmet need for safe, non-invasive therapeutic strategies.

**Case presentation:**

A 30-year-old primigravida at 16 weeks of gestation presented with a giant (>15 cm), antibiotic-refractory left breast PCM. She declined systemic corticosteroids and surgical intervention due to fetal safety concerns. An individualized three-stage topical herbal residue dressing regimen was implemented, with dynamic prescription adjustments from mass resolution to wound healing to postpartum consolidation. Oral herbal medicine was added postpartum when fetal safety was no longer a constraint.

**Results:**

Clinical cure was achieved, defined as complete resolution of the palpable mass and ulcer healing. A notable discordance between clinical and imaging recovery was observed, with residual hypoechoic areas requiring over 1 year to achieve near-complete resolution. The patient remained recurrence-free throughout a 3-year follow-up period, including a 2-year treatment-free phase. A healthy full-term infant was delivered vaginally with normal neonatal development.

**Conclusion:**

This case suggests that staged topical herbal therapy may represent a potential option for managing gestational PCM in carefully selected patients. These findings may serve as a reference for clinicians managing similar cases but require confirmation through larger controlled studies before definitive clinical recommendations can be made.

## 1 Introduction

PCM is a chronic inflammatory breast disease belonging to the category of non-lactational mastitis, with core pathological features of mammary duct ectasia and dense plasma cell infiltration in the lesion (1–4). The etiology of PCM remains not fully elucidated, and the disease is clinically characterized by high recurrence rate, frequent postoperative complications, and limited efficacy of conventional treatments, bringing great challenges to clinical management (2, 3).

For pregnant patients with PCM, hormonal fluctuations may influence disease progression and increase the complexity of diagnosis and treatment (5). More importantly, clinical decision-making for gestational PCM must balance the dual needs of effective disease control and fetal safety. Conventional first-line treatments for non-gestational PCM, including glucocorticoids, anti-tuberculosis drugs, and surgical resection, all have clear limitations in pregnancy, with non-negligible risks of adverse maternal-fetal outcomes (1, 3, 6). To date, published studies on safe and effective therapeutic regimens for gestational PCM are very scarce, and there is no standardized clinical guideline for this special population, leading to a prominent clinical dilemma.

We report a 30-year-old primigravida at 16 weeks of gestation who presented with a 1-month history of progressive left breast redness, swelling, and severe pain, accompanied by fever and arthralgia. Physical examination revealed a 15 × 5 cm hard mass in the left breast. Although core needle biopsy confirmed PCM, the lesion progressed despite standard antibiotic therapy. Given the patient’s refusal of systemic medication due to fetal safety concerns, she was treated with an individualized staged topical herbal residue dressing regimen. Clinical cure was achieved with a favorable obstetric outcome, and the patient remained recurrence-free throughout a 3-year follow-up period. This case adds to the limited evidence on the potential role of topical herbal therapy in managing gestational PCM.

## 2 Case presentation

### 2.1 Patient information

A 30-year-old primigravida at 16 weeks of gestation presented with a 1-month history of progressive left breast redness, swelling, severe pain, fever, and arthralgia. She had previously received a 1-week course of intravenous antibiotics at a local hospital without symptom relief. Given her pregnancy, she declined systemic corticosteroid therapy and surgical intervention, and was therefore referred to our institution for further management. She had no history of breast disease, autoimmune disorder, chronic illness, or adverse pregnancy outcomes. She denied known drug allergies.

### 2.2 Clinical findings

On admission, physical examination revealed a firm, tender 15 × 5 cm mass in the upper outer quadrant of the left breast, with no overlying skin changes, nipple discharge, or axillary lymphadenopathy.

### 2.3 Diagnostic assessment

Initial ultrasound at the outside hospital demonstrated multiple low-echo lesions in the left upper outer quadrant, with the largest showing irregular shape, unclear margins, uneven internal echoes, and rich blood flow on Doppler. Enlarged reactive lymph nodes were noted in the left axilla and supraclavicular region. The lesion was assessed as BI-RADS 4A, favoring an inflammatory process. The right breast was BI-RADS 1.

A core needle biopsy confirmed PCM, revealing lymphoplasmacytic infiltration with ductal dilatation and periductal inflammatory change, without evidence of malignancy. This definitive histopathological diagnosis ruled out key differentials including idiopathic granulomatous mastitis, inflammatory breast cancer, and breast tuberculosis (3, 7, 8).

### 2.4 Therapeutic intervention

Given the pregnancy, maternal–fetal safety was prioritized. The patient declined systemic medication and surgery. An individualized three-stage topical herbal residue regimen was implemented, with herbs selected according to pharmacopoeial pregnancy safety guidance. The exclusive topical route minimized systemic exposure.

Stage 1 (gestational week 16): External application of Wentong Xiaoyong Formula, twice daily for 30 min.

Stage 2 (gestational week 24): After ulceration developed, the prescription was adjusted by removing potent blood-activating herbs and adding dampness-drying and astringent herbs to promote wound healing.

Stage 3 (1 month postpartum): With lactation completed and fetal safety concerns resolved, oral Chinese medicine was added to the topical regimen for consolidation.

The complete composition and stage-specific adjustments of the herbal regimen are presented in Table 1.

**Table 1:** Composition of the three-stage herbal regimen.

No.	Herb (Latin Name)	Herb (English Name)	2022.02.23 (Stage 1)	2022.04.24 (Stage 2)	2022.09.06 (Stage 3)	2022.11.22	2023.02.14 (clinical cure)
	Topical	Topical	Topical + oral	Topical + oral	Topical	Topical + oral	Topical
1	Clematidis Armandii Caulis	Armand Clematis Stem	15	15	—	—	15
2	Tribuli Fructus Tostus	Stir-fried Puncturevine Caltrop Fruit	15	15	15	15	15
3	Bombycis Batryticatus Tostus	Stir-fried Stiff Silkworm	15	15	—	15	15
4	Prunellae Spica	Selfheal Spike	15	15	15	15	15
5	Salviae Chinensis Herba	Chinese Sage Herb	15	15	15	15	15
6	Liquidambaris Fructus	Sweetgum Fruit	10	10	10	10	10
7	Artemisiae Argyi Folium	Argy Wormwood Leaf	10	10	—	—	10
8	Sinapis Semen Tostus	Stir-fried Mustard Seed	10	10	—	—	10
9	Citri Reticulatae Semen Salinus	Salt-fried Tangerine Seed	10	—	10	10	10
10	Litchi Semen Salinus	Salt-fried Lychee Seed	10	—	10	10	—
11	Sonchi Arvensis Herba	Field Sow-thistle Herb	15	15	15	15	15
12	Sparganii Rhizoma Aceticum	Vinegar-processed Sparganium Rhizome	15	—	—	15	15
13	Curcumae Rhizoma Aceticum	Vinegar-processed Turmeric Rhizome	15	—	—	15	15
14	Hedyotidis Diffusae Herba	Spreading Hedyotis Herb	15	15	—	—	15
15	Luffae Fructus Retinervus	Membranous Luffa Fruit	6	6	—	—	—
16	Mori Ramulus	Mulberry Twig	15	—	—	—	—
17	Sophorae Flavescentis Radix	Knotweed Resurrection Lily Root	—	10	—	—	—
18	Cnidii Fructus	Cnidium Fruit	—	15	15	—	15
19	Kochiae Fructus	Burning Bush Fruit	—	15	—	—	—
20	Natrii Sulfas	Sodium Sulfate	—	20	—	—	—
21	Chebulae Fructus	Terminalia Fruit	—	10	—	—	—
22	Smilacis Glabrae Rhizoma	Glabrous Greenbrier Rhizome	—	—	15	15	—
23	Dioscoreae Hypoglaucae Rhizoma	Ground Orchid Tuber	—	—	20	20	—
24	Alismatis Rhizoma	Alisma Rhizome	—	—	15	—	—
25	Bolbostemmatis Rhizoma	Snake Lily Bulb	—	—	10	10	—
26	Cremastrae Pseudobulbus	Jewel Orchid Bulb	—	—	10	—	—
27	Forsythiae Fructus	Forsythia Fruit	—	—	15	15	—
28	Phellodendri Amurensis Cortex	Amur Cork Tree Bark	—	—	15	15	—
29	Achyranthis Bidentatae Radix	Twin-slice Achyranthes Root	—	—	15	15	—
30	Polyporus	Polyporus	—	—	15	—	—
31	Salviae Miltiorrhizae Radix	Red Sage Root	—	—	15	15	—
32	Hordei Fructus Germinatus Praeparatus	Scorched Barley Sprout	—	—	30	—	—
33	Euryales Semen Praeparatum	Stir-fried Euryale Seed	—	—	15	15	—
34	Polygonati Rhizoma Vinum	Wine-processed Solomon’s Seal Rhizome	—	—	10	10	—
35	Rhodiolae Crenulatae Radix	Crenulate Rhodiola Root	—	—	10	10	—
36	Carthami Flos	Safflower	—	—	—	—	15
37	Hirudo Praeparatus	Prepared Leeches	—	—	—	—	6
38	Ostreae Concha (decocted first)	Oyster Shell (decocted first)	—	—	—	30	—
39	Arcae Concha (decocted first)	Calcined Clam Shell (decocted first)	—	—	—	6	—

This table presents the active treatment period (February 2022–February 2023). The regimen was dynamically adjusted according to disease evolution. All herbs used during pregnancy are classified as safe for gestational use. Potent blood-activating herbs were strictly time-limited and discontinued immediately upon achieving the therapeutic goal. Detailed safety considerations are discussed in the Discussion section. This table presents the herbal regimen during the active treatment period (February 2022–February 2023). The regimen was dynamically adjusted according to disease evolution and gestational/postpartum status.

Stage 1 (2022.02.23, gestational week 16): The core formula was applied topically. Given the acute, rapidly progressive giant inflammatory mass unresponsive to antibiotics, potent blood-activating and mass-resolving herbs were included to address the clinical urgency. These herbs were used for a strictly limited duration of 8 *weeks*.

Stage 2 (2022.04.24, gestational week 24): After successful mass softening and ulceration, the treatment priority shifted to wound healing. Blood-activating herbs were removed, and dampness-drying, heat-clearing, and astringent herbs were added to control local inflammation and promote wound closure.

Stage 3 (2022.09.06, 1 month postpartum): With fetal safety no longer a concern, oral Chinese medicine was added to the topical regimen to target deeper residual inflammation and support postpartum recovery.

### 2.5 Follow-up and outcomes

The patient continued standardized home-based topical therapy throughout the remainder of her pregnancy. She delivered a healthy full-term infant vaginally. At 1 month postpartum, the wound was nearly healed, though a large residual mass remained palpable. Ultrasound at this time revealed a persistent deep inflammatory lesion with rich blood flow, despite superficial wound closure.

On February 14, 2023, the breast mass had completely resolved on palpation and the ulcer was fully healed, indicating clinical cure. However, ultrasonography still showed a large residual area, albeit with markedly reduced vascularity and relatively clear margins. This discordance between clinical resolution and persistent imaging abnormalities is a recognized phenomenon in inflammatory breast conditions. Active treatment was discontinued at this point, and the patient was transitioned to long-term surveillance. Intermittent herbal applications continued until April 2024 for scar remodeling and recurrence prevention, not for active disease treatment.

Serial ultrasonography over the following year documented gradual and progressive spontaneous absorption of the residual lesion, with continuous decreases in both lesion size and blood flow.

At the 3-year follow-up in February 2026, bilateral breast architecture had largely normalized. A small, well-circumscribed residual nodule remained, assessed as BI-RADS 3. No disease recurrence was observed following the cessation of all therapy in April 2024, representing a 2-year treatment-free recurrence-free period. Throughout the entire follow-up from clinical cure (February 2023) to the final visit (February 2026), the patient remained asymptomatic with no evidence of disease reactivation.

### 2.6 Timeline

**Figure 1:**
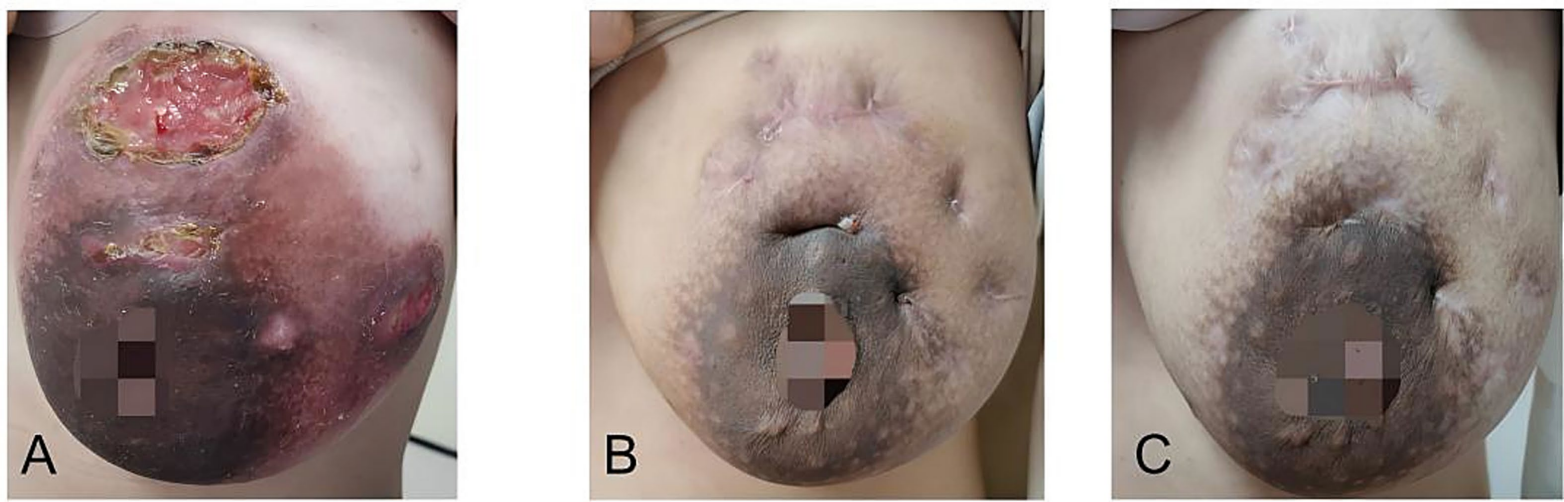
Local manifestations of the left breast lesion at different treatment stages. **(A)** April 24, 2022 (gestational week 24, ulcerative stage): a 5 × 4 cm area of ulceration with multiple punctate openings on the upper aspect of the left breast. **(B)** September 6, 2022 (1 month postpartum, consolidation stage): the ulcerated wound was largely healed, with residual granulomatous tissue visible at the site. **(C)** February 14, 2023 (18-month follow-up): the wound was completely healed, the scar had become markedly lighter and softer, and clinical cure was achieved.

A comprehensive timeline of interventions, clinical changes, and ultrasonographic findings is presented in Table 2. Representative clinical images are shown in Figure 1.

**Table 2:** Timeline of clinical course, therapeutic interventions, and imaging findings.

Time point	Gestational/postpartum stage	Key clinical events	Therapeutic interventions	Core disease changes	Key ultrasonographic findings
Jan 2022	12 weeks of gestation	First onset of left breast redness, pain, fever, and arthralgia	1-week IV antibiotics at outside hospital	No symptom relief, progressive enlargement of the breast mass	Baseline (outside hospital): Left breast BI-RADS 4A. Multiple low-echo lesions; largest 5.0 × 1.5 cm at 11 o’clock, irregular, unclear margins, rich blood flow. Reactive lymph nodes in left axilla (2.8 × 0.8 cm) and supraclavicular region (1.8 × 0.9 cm). Right breast BI-RADS 1.
Feb 23, 2022	16 weeks of gestation	Confirmed diagnosis of PCM via core needle biopsy at outside hospital, transferred to our hospital	Stage 1: External application of Wentong Xiaoyong Formula, twice a day, 30 min each time	15 × 5 cm hard mass in the left breast, no ulceration	Not performed during admission (baseline imaging already available).
Apr 24, 2022	24 weeks of gestation	Ulceration and exudation of the left breast mass, complete resolution of arthralgia	Stage 2: Adjusted topical prescription, removed blood-breaking herbs, added dampness-drying and astringent herbs	Mass softened; a 5 × 4 cm ulceration developed; systemic symptoms resolved	Not performed (clinical monitoring prioritized to minimize fetal ultrasound exposure).
May 2022 - Delivery	25 weeks of gestation to full-term delivery	Standardized home-based topical treatment, regular prenatal check-ups	Continuous topical treatment with the adjusted prescription from Stage 2	Ulcerated wound gradually healed; normal fetal development; uneventful full-term vaginal delivery; healthy neonate	Not performed.
Sep 6, 2022	1 months postpartum	Completed lactation cessation, the wound was basically healed with residual granulomatous changes	Stage 3: Adjusted to combined oral and topical consolidation treatment	Wound healing achieved; a large residual mass remained palpable	Left breast: diffuse confluent hypoechoic areas, ~11.0 × 10.0 × 4.5 cm, relatively clear borders, irregular shape, rich blood flow, internal liquefaction, tunnel-like extension.
Feb 14, 2023	18 months postpartum	Clinical cure documented	Discontinued active treatment; intermittent topical herbs for scar remodeling	Complete resolution of breast mass on palpation, full healing of ulcer, shallowing of scar	Left breast: residual extensive hypoechoic areas, ~10.0 × 10.0 × 2.8 cm, relatively clear margins, minimal blood flow, internal liquefaction, tunnel-like extension.
May 16, 2023	21 months postpartum	Regular ultrasound follow-up	Intermittent topical herbs for scar remodeling	No abnormal physical signs	Left breast: residual hypoechoic area reduced to ~10.0 × 2.0 cm, ill-defined margins, irregular shape, minimal blood flow.
Oct 10, 2023	27 months postpartum	Regular ultrasound follow-up	Intermittent topical herbs for scar remodeling	No abnormal physical signs	Left breast: residual hypoechoic area further reduced to ~6.3 × 1.3 cm, ill-defined margins, irregular shape, minimal blood flow, local extension to subcutaneous tissue.
Apr 30, 2024	/	Last herbal application	Cessation of all herbal therapy	No abnormal physical signs	Ultrasound not performed at this visit; clinical examination only
Feb 2026	3-year follow-up	Final evaluation	/	No disease recurrence, good recovery of breast tissue	Left breast: 0.5 × 0.4 cm low-echo nodule at 12 o’clock, clear margins, BI-RADS 3. Right breast: no definite mass. Bilateral breast architecture largely normalized.

IV, intravenous; PCM, plasma cell mastitis; BI-RADS, Breast Imaging Reporting and Data System. Herbal applications after February 14, 2023 (clinical cure) were administered for scar remodeling and recurrence prevention, not for active disease. All therapy was permanently discontinued on April 30, 2024. No disease recurrence was observed during the subsequent 2-year treatment-free follow-up.

## 3 Discussion

### 3.1 Summary of key findings

This case report describes the management of a giant (>15 cm), antibiotic-refractory PCM in a 30-year-old primigravida using a staged topical herbal dressing regimen. Clinical cure was achieved by February 2023, with complete resolution of the palpable mass and ulcer healing. A discordance between clinical and imaging recovery was observed in which residual hypoechoic areas required over 1 year to achieve near-complete resolution. The patient remained recurrence-free throughout a 3-year follow-up period, including a 2-year treatment-free phase, culminating in sustained clinical and imaging remission by February 2026.

### 3.2 Clinical implications and relationship to existing literature

The management of gestational PCM presents a significant clinical dilemma. While short-term glucocorticoids may alleviate inflammation, recent guidelines caution against their use during pregnancy owing to dose-dependent risks including preterm birth and fetal growth restriction (6, 9, 10). The safety of anti-tuberculosis drugs in this context remains undefined, and surgical intervention carries unacceptable miscarriage risks (11, 12). Compared with existing literature, this is, to our knowledge, the first report of giant gestational PCM managed entirely with topical herbal therapy, thereby avoiding systemic fetal pharmacological exposure (13, 14). It also provides robust long-term follow-up data, contrasting with the high relapse rates reported for conventional surgical approaches (15).

The observed clinical-imaging discordance is a key finding. This phenomenon, likely reflecting the slow absorption of inflammatory exudates and fibrotic remodeling, is well recognized in inflammatory breast conditions but remains underappreciated in PCM (16). Its clinical significance is twofold: it highlights that imaging endpoints should not be used in isolation to judge treatment failure, and it underscores the need for prolonged surveillance to avoid premature intervention.

### 3.3 Rationale and possible mechanisms of action

The rationale for the topical regimen is rooted in the TCM principle that external treatment follows the same therapeutic logic as internal treatment, as articulated in the classic text Liyue Pianwen (17). Although the precise pharmacological mechanisms remain to be fully elucidated, several hypotheses may explain the observed effects.

First, the warm herbal dressing may improve local microcirculation, facilitating inflammatory exudate absorption and tissue repair (18–20). Second, bioactive compounds such as flavonoids and alkaloids present in the prescribed herbs may exert local anti-inflammatory effects through transdermal delivery (21, 22). A recent study demonstrated that immune factor expression correlates with surgical prognosis in PCM, suggesting that immunomodulation may be a relevant therapeutic target (23). The clinical improvement observed may partly result from the immunomodulatory properties of the applied herbs.

The three-stage prescription adjustments align with the evolving pathophysiology. Stage 1 employed potent blood-activating and mass-resolving herbs to address the acute, rapidly enlarging inflammatory mass. After ulceration developed in Stage 2, ongoing fibrinolytic activity could be counterproductive to wound closure, providing the rationale for discontinuing blood-activating herbs. The herbs introduced at this stage possess known anti-inflammatory and astringent properties that may facilitate wound healing (24, 25). Stage 3, initiated postpartum, incorporated oral herbal medicine with deeper anti-inflammatory and tissue-repairing properties to eliminate residual lesions.

### 3.4 Safety considerations

All herbs used during pregnancy were selected in strict accordance with the pregnancy medication specifications of the Pharmacopoeia of the People’s Republic of China (2025 Edition) (26). Topical administration minimized systemic absorption, consistent with the generally limited transdermal bioavailability of herbal compounds (27, 28). The uncomplicated full-term vaginal delivery and normal neonatal development provide preliminary clinical corroboration, though formal pharmacokinetic studies during pregnancy are lacking.

The inclusion of blood-activating herbs (Sparganii Rhizoma, Curcumae Rhizoma) in Stage 1 was guided by a risk–benefit assessment in which the threat posed by a rapidly enlarging, antibiotic-refractory giant inflammatory mass was considered to outweigh the risks of short-term topical exposure. These herbs were discontinued immediately once the therapeutic goal was achieved in Stage 2, limiting exposure to 8 weeks.

### 3.5 Limitations

This study has several important limitations. First, as a single case report, no causal inferences can be drawn, and the findings are not generalizable. The observed therapeutic effect may partly reflect the natural history of PCM, and the contribution of the herbal intervention cannot be isolated without a controlled design. Second, serial ultrasonography during pregnancy was not performed to minimize fetal exposure, resulting in a gap in imaging documentation during the treatment period. Third, the herbal preparations were manually compounded, limiting standardization and reproducibility. Fourth, formal pharmacokinetic and pharmacodynamic data are lacking, and the proposed mechanisms should therefore be considered hypothetical.

### 3.6 Conclusions and lessons learned

For pregnant patients with giant PCM refractory to antibiotics who decline conventional systemic therapy, staged topical herbal dressing may represent a potential option in carefully selected cases. In this instance, the treatment was associated with local disease control, sustained long-term remission, and a favorable pregnancy outcome, without evidence of harm to the mother or fetus. The key lesson from this case is that for inflammatory breast conditions, clinical cure and imaging resolution may follow distinct timelines; extended imaging surveillance is necessary to avoid premature declaration of treatment failure. Controlled prospective studies are required before this approach can be considered a reliable clinical strategy.

## 4 Patient perspective

After being diagnosed with PCM at 16 weeks of gestation, the patient was advised to undergo surgery and receive systemic medications at the referring hospital. She declined these interventions due to concerns about potential adverse effects on fetal development and was transferred to our institution for alternative management.

Following admission, she received the individualized topical herbal dressing regimen. During treatment, she noted gradual improvement of the breast mass and relief of local pain, and reported that her initial anxiety progressively resolved as her condition improved. She expressed satisfaction with the therapeutic outcome and particularly valued the avoidance of surgical trauma and systemic medication during pregnancy.

## Data Availability

The datasets presented in this article are not readily available because the data supporting this case report cannot be made publicly available due to patient privacy and ethical restrictions. Requests to access the datasets should be directed to Xiaojun Zhang, zxj6298@sohu.com.

## References

[1] Das Sheth A Joshi S Kumar A Nair N Shet T Sahay A . Management of idiopathic granulomatous mastitis: effectiveness of a steroid-free regimen using *Tinospora cordifolia*-a single-institution experience. Breast J. (2025) 2025:2997891. doi: 10.1155/tbj/2997891, 39886361 PMC11779988

[2] Zhou Y Gong J Deng X Shen L Liu L. Novel insights: crosstalk with non-puerperal mastitis and immunity. Front Immunol. (2024) 15:1431681. doi: 10.3389/fimmu.2024.1431681, 39148739 PMC11324573

[3] Zhou F Shang X-C Tian X-S Yu Z-G Chinese Society of Breast Surgery. Clinical practice guidelines for diagnosis and treatment of patients with non-puerperal mastitis: Chinese Society of Breast Surgery (CSBrS) practice guideline 2021. Chin Med J. (2021) 134:1765–7. doi: 10.1097/CM9.0000000000001532, 34039865 PMC8367070

[4] Mitchell KB Valente SA Snider HC Fowler AM Allison KH Pass HA . American Society of Breast Surgeons, Society of Breast Imaging, and College of American Pathology 2025 guidelines for the Management of Infectious and Inflammatory Lesions of the breast. JAMA Surg. (2026). doi: 10.1001/jamasurg.2026.0613, 41920556

[5] Ajmal N Ma LX Palazzo JP. The pathologic spectrum of pregnancy- and lactation-associated breast lesions. Arch Pathol Lab Med. (2025) 149:1033–41. doi: 10.5858/arpa.2024-0461-RA, 40151997

[6] Gyamfi-Bannerman C Clifton RG Tita ATN Blackwell SC Longo M de Voest JA . Neurodevelopmental outcomes after late preterm antenatal corticosteroids. JAMA. (2024) 331:1629–37. doi: 10.1001/jama.2024.4303, 38656759 PMC11044009

[7] Tian C Han X Liu Z Lv X Ning P. Management of granulomatous lobular mastitis and risk factors associated with recurrence. World J Surg. (2022) 46:2706–14. doi: 10.1007/s00268-022-06687-7, 35963955

[8] Kang W Zhang X-J. Inflammatory breast cancer was misdiagnosed as plasma cell mastitis: a case report. Asian J Surg. (2023) 46:1153–4. doi: 10.1016/j.asjsur.2022.08.020, 36050239

[9] Pluma A Hamroun S Rüegg L Cecchi I Kramer M Perez-Garcia LF . Antirheumatic drugs in reproduction, pregnancy, and lactation: a systematic literature review informing the 2024 update of the EULAR recommendations. Ann Rheum Dis. (2025) 84:1561–90. doi: 10.1016/j.ard.2025.02.021, 40240264

[10] Rüegg L Pluma A Hamroun S Cecchi I Perez-Garcia LF Anderson PO . EULAR recommendations for use of antirheumatic drugs in reproduction, pregnancy, and lactation: 2024 update. Ann Rheum Dis. (2025) 84:910–26. doi: 10.1016/j.ard.2025.02.023, 40287311

[11] Hughes J. Pharmacokinetics and safety of group A and B anti-tuberculosis drugs used in treatment of rifampicin-resistant tuberculosis during pregnancy and post-partum: a narrative review. Pathogens. (2023) 12:1385. doi: 10.3390/pathogens12121385, 38133270 PMC10745846

[12] AkbariRad M Sheybani F Firoozi A Sajjadi S Emadzadeh M Kazerani M . Treatment outcomes of lobular granulomatous mastitis: impact of hyperprolactinemia, diabetes, and erythema nodosum-insights from a 7-year cohort study. Breast J. (2026) 2026:e2672157. doi: 10.1155/tbj/2672157, 41871228 PMC13140925

[13] Awomolo AM Louis-Jacques A Crowe S. Idiopathic granulomatous mastitis diagnosed during pregnancy associated with successful breastfeeding experience. BMJ Case Rep. (2021) 14:e241232. doi: 10.1136/bcr-2020-241232, 34413030 PMC8378369

[14] Yoshino R Yoshida N Ito A Ujiie N Nakatsubo M Hayashi M . Granulomatous mastitis occurring during pregnancy: a case report. Medicina (Kaunas). (2023) 59:1418. doi: 10.3390/medicina59081418, 37629708 PMC10456481

[15] Wang P Li L Liu Y Shi Y Zhang X Wu J. Targeted duct excision reduces recurrence and shortens treatment duration in periductal mastitis: a retrospective cohort study. Int J Women's Health. (2026) 18:583120. doi: 10.2147/IJWH.S583120, 41756563 PMC12932089

[16] Ozcan BB Merchant K Goldberg J Burns Z Sahoo S Compton L . Can imaging findings predict the outcome of idiopathic granulomatous mastitis? Ann Breast Surg. (2023) 7:35. doi: 10.21037/abs-22-23

[17] Wu S. Li Yue Pian Wen. Beijing: China Medical Science Press (2018).

[18] Li T Zhang L Qu X Lei B. Advanced Thermoactive nanomaterials for thermomedical tissue regeneration: opportunities and challenges. Small Methods. (2025) 9:e2400510. doi: 10.1002/smtd.202400510, 39588862

[19] Liu Q Zhang XJ. Zhang Xiaojun jiaoshou zhongyao waifu zhiliao yunqi jiangxibao ruxianyan jingyan [Professor Zhang Xiaojun’s experience in treating plasma cell mastitis during pregnancy with external application of Chinese herbal medicine]. Zhongguo Yiyao Daobao. (2023) 20:146–9. doi: 10.20047/j.issn1673-7210.2023.31.31

[20] Kaur R Arora S Goswami M. Formulation development and evaluation of transdermal patch of astaxanthin. Mater Today Proc. (2022). doi: 10.1016/j.matpr.2022.11.119, 38826717

[21] Lin C-F Lin M-H Hung C-F Alshetaili A Tsai Y-F Jhong C-L . The anti-inflammatory activity of flavonoids and alkaloids from *Sophora flavescens* alleviates psoriasiform lesions: prenylation and methoxylation beneficially enhance bioactivity and skin targeting. Phytother Res. (2024) 38:1951–70. doi: 10.1002/ptr.8140, 38358770

[22] Kim HP. The long search for pharmacologically useful anti-inflammatory flavonoids and their action mechanisms: past, present, and future. Biomol Ther (Seoul). (2022) 30:117–25. doi: 10.4062/biomolther.2022.004, 35131949 PMC8902448

[23] Li S Lin X. Correlation between immune factor expression and prognosis of plasma cell mastitis surgery and its predictive value for prognosis. Clin Exp Obstet Gynecol. (2024) 51:234. doi: 10.31083/j.ceog5110234

[24] Li W Gao J Wang M Xie Z Guo Z Chen Z . Discovery of antibacterial active components from the root barks of Sophora flavescens and evaluation of their antibacterial in vitro and infected wound healing activity in vivo. J Ethnopharmacol. (2026) 366:121617. doi: 10.1016/j.jep.2026.121617, 41933752

[25] Xu P Wang A Xiao Z Song W Pan H Yang J . Osthole promotes skin flap survival by inhibiting ferroptosis and alleviating pyroptosis. J Ethnopharmacol. (2026) 358:121058. doi: 10.1016/j.jep.2025.121058, 41406561

[26] Chinese Pharmacopoeia Commission. Pharmacopoeia of the People’s Republic of China. 2025th ed. Beijing: China Medical Science Press (2025).

[27] Neupane R Boddu SHS Abou-Dahech MS Bachu RD Terrero D Babu RJ . Transdermal delivery of chemotherapeutics: strategies, requirements, and opportunities. Pharmaceutics. (2021) 13:960. doi: 10.3390/pharmaceutics13070960, 34206728 PMC8308987

[28] Cheng Y-C Li TS Su HL Lee PC Wang H-MD. Transdermal delivery systems of natural products applied to skin therapy and care. Molecules. (2020) 25:5051. doi: 10.3390/molecules25215051, 33143260 PMC7662758

